# Splenic Artery Aneurysm (SAA) Rupture in Pregnancy: A Case Report of a Rare but Life-Threatening Obstetrical Complication

**DOI:** 10.26502/fjwhd.2644-2884004

**Published:** 2019-05-20

**Authors:** Rami A Ballout, Rayan Ghanem, Anwar Nassar, Ali H Hallal, Labib M Ghulmiyyah

**Affiliations:** 1 Faculty of Medicine, American University of Beirut, Beirut, Lebanon; 2 Department of Obstetrics and Gynecology, American University of Beirut Medical Center, Beirut, Lebanon; 3 Department of Surgery, American University of Beirut Medical Center, Beirut, Lebanon

**Keywords:** Spleen, Splenic rupture, Hemoperitoneum, Maternal, Fetal, Obstetrics

## Abstract

This is the case of a 38 year-old Lebanese woman G2P1, history of previous cesarean section, presenting at 30+5 weeks of gestation with acute left-sided flank pain and a two-day history of chills and dysuria. In light of the clinical presentation, the patient was initially diagnosed with pyelonephritis and managed accordingly; however, her clinical status deteriorated with worsening hypotension and lethargy despite resuscitative measures and a normal abdominal ultrasound. Failure to revive the patient eventually led to a cardiac arrest for which a peri-mortem cesarean section was performed at bedside. Upon abdominal entry, an actively-bleeding ruptured splenic artery aneurysm (SAA) was identified, for which massive transfusion protocol was activated, and the patient was transferred to the operating room. The patient had a complicated postoperative course, the fetus was stillborn, and she was discharged home after 6 months of hospital stay. In view of the high mortality and morbidity associated with ruptured SAA in pregnancy, early recognition and prompt intervention are crucial for maternal and fetal benefit.

## 1. Introduction

A wise man once said “For an understanding of the future, look to the past” [1]. Outside of pregnancy, SAA was first reported by Beaussier in 1770, and another case was then reported by Parker in 1844; these two cases were for many years omitted from the literature not to mention erroneously credited to another author [2]. Since Beaussier and until about 1956, only 213 cases of SAA had been reported in the literature [2]. In 1903, Winkler was the first to identify SAA in a living person. In 1920, Hoegler, made the first preoperative diagnosis clinically. In 1932, the first diagnosis to be based on radiological examination alone was made by Lindboe, and in 1950, Evans obtained the first translumbar aortogram demonstrating a SAA; the subject was then extensively reviewed by Owens and Coffey in 1953 [2]. By 1960, around 250 cases of splenic artery aneurysm were reported in the literature with an incidence at the time of 10.4% and with medial degeneration being attributed as the most common etiological factor [2]. Since the 1900’s, we have come a long way and at least 400 cases of SAA have been described throughout history, nonetheless, the entity is still regarded vague in its entirety. Splenic artery aneurysms are the third most common true aneurysm, after aortic and iliac artery aneurysms, representing 60% of visceral artery aneurysms [3]. In the general population, the prevalence of SAA is less than 1%, and it is more common in women, with a female: male ratio of 4:1. Splenic artery aneurysms usually present in the 5^th^ and 6^th^ decade of life with a mean age of presentation at 52 years, and with as many as 80% occurring in patients above the age of 50 [3]. It should be noted that the prevalence of SAA in females of childbearing age is less than 0.1% [3]. Splenic artery aneurysm specific risk factors include a varied entity ranging from arterial medial fibrodysplasia that entails renal artery stenosis and secondary hypertension, as well as any hepatic, splenic, or even pancreatic pathology [3]. Splenic artery aneurysm rupture during pregnancy, a rare but serious obstetrical complication, was first reported in the literature by the Danish obstetrician Sylver Saxtorph in 1803 [4].

In 1912, Wesenberg reported on 10 pregnant women with ruptured SAA during the third trimester that resulted in 8 maternal deaths, where Wesenberg concluded that the rupture was attributed to the pregnancy itself [5]. According to many sources, pregnancy is considered as one of the most significant risk factors for the rupture of a splenic artery aneurysm [3]; the impact that pregnancy has on the pathogenesis of SAA should never be underestimated. In a systemic review on SAA in pregnancy, different mechanisms were proposed in its pathogenesis [6]. Trimble and Hill’s proposals were first described in the 1942, and they suggested that aneurysmal dilatation of an artery results from two contributing factors: weakness in the arterial wall and an increase in blood pressure [6], amongst these physiological changes, an increased blood volume and cardiac output result in increased blood flow, and ultimately increased mechanical resistance against the vessel wall leading to its weakening [6]. Other physiological changes in pregnancy, further compounding the risk of splenic artery aneurysm rupture, include an increased estrogen and progesterone that are thought to cause medial degeneration of the vessel wall, as well as elevated levels of relaxin thought to enhance the elasticity of the splenic artery leading to aneurysmal dilation [6–9]. It was reported that 50% of SAA ruptures occur in pregnant women [10], with 95% of all pregnancy-associated SAAs being identified secondary to rupture; the ruptures occur predominantly in multiparous women, 40% of whom are grand multiparous [10].

The highest risk of rupture for a splenic artery aneurysm is during the third trimester, accounting for 69% of ruptures, typically in the last two weeks of pregnancy; 12% of ruptures occur during the 1^st^ and 2^nd^ trimesters, and 13% occur during labor with 6% occurring postpartum [10–13]. There are many different faces to how a splenic artery aneurysm can present in pregnancy, and more often the presentation is significantly misleading: True aneurysms can be silent and asymptomatic; whereas, pseudo aneurysms are always symptomatic. Patients with SAA are more often asymptomatic, with only 20% presenting with symptoms, mainly nonspecific complaints including vague epigastric or left upper quadrant abdominal pain radiating to the left shoulder [14]. The classic presentation of a ruptured splenic artery aneurysm occurs in 25% of cases [15]. The latter, which is known as the double rupture phenomenon occurs in two stages: at first, the initial hemorrhage is limited when blood clots block the foramen of Winslow, temporarily containing the hemorrhage to the lesser sac that is followed 6–96 hours later by free rupture into the peritoneal cavity with hemodynamic instability, that can lead to sudden maternal and fetal death [15]. The benefit of the two-stage rupture is that it can allow time for effective diagnosis and treatment [15]. The risk of splenic artery aneurysm rupture in pregnancy is 2–3%, where the incidence of rupture is 3.6 occurrences per 100,000 pregnancies [16]. Of importance is the significant difference in mortality between pregnant and non-pregnant patients: mortality outside of pregnancy ranges from 10–25%, whereas in pregnancy, maternal mortality increases up to 75%, and fetal mortality increases to as high as 95% [16]. The highest risk of rupture is during pregnancy and unfortunately, despite the best efforts, often leads to a loss of two lives; Of the 400 reported cases on splenic artery aneurysm in pregnancy, only 12 have been reported with the survival of both mother and fetus [17]. It goes without saying that the risk of rupture appears to be strongly associated with the diameter of the lesion, demonstrating a linear relationship [16]. The size of the aneurysm is usually greater than 2.5 cm in most patients at the time of rupture; nonetheless, proactive management is usually implemented at the time of pregnancy, as the diameter of the aneurysm does not reflect the probability of rupture [6, 16].

Moreover, even giant splenic artery aneurysms have been reported in the literature [18]. In the majority of cases, the clinical signs of a ruptured SAA in pregnancy are masked and it is a commonly misdiagnosed entity. 70% of cases of SAA rupture during pregnancy are misdiagnosed as uterine rupture [19, 21–23], placental abruption is another one of the most commonly made misdiagnosis [19]. The majority of splenic artery aneurysms are diagnosed at the time of laparotomy; nonetheless, in the setting of a high clinical suspicion, radiological investigations are helpful in making the diagnosis in both emergency and elective settings; the diagnostic utility of radiologic studies, however, in pregnancy remains questionable [24]. Moreover, they should not delay the immediate resuscitation and control of the hemorrhage by emergency surgery [24]. Abdominal imaging examination carried out for unrelated disorders may raise suspicion of a splenic artery aneurysm that appears as a classic calcified ring in the left upper abdominal quadrant on a plain x-ray of the abdomen. Calcification, which is often viewed as evidence of chronicity and stability, was reported in one study to occur in 90% of the ruptured aneurysms, and thus, its prognostic value is not clear [24].

Digital subtraction angiography was in the past considered the “gold standard” for the diagnosis of vascular diseases, being considered the most specific imaging test to identify SAAs with added therapeutic benefits [25]. With the advent of noninvasive imaging technologies, especially the development of multi slice CT angiography imaging technology, diagnostic accuracy reached its highest [25]. The ultrasound is the preferred initial imaging modality for identifying splenic artery aneurysm in pregnancy due to its noninvasiveness and relative safety profile [26]. During pregnancy, diagnosis of both an unruptured and ruptured SAA is best performed by US with pulsed Doppler. Nonetheless, the utility is limited by operator dependency, obese patients, bowel gas shadowing and arteriosclerosis, amongst others [26]. In short, because of the high mortality rate and common misdiagnosis, splenic artery aneurysm should be on the differential of any pregnant woman with abdominal pain, especially in the setting of hemodynamic instability [20].

## 2. Case Study

A 38 year-old G2P1 presented at 30+5 weeks of gestation, to the emergency department, with sudden onset of severe left flank pain. Review of systems was significant for chills and dysuria. On examination, the patient’s abdomen was soft, non-tender, with no guarding or rebound tenderness, but with prominent left costovertebral angle tenderness. Laboratory workup at the time included a complete blood count and serum chemistries that were unremarkable. A FAST scan in the emergency department was performed for further evaluation, with no remarkable findings. Accordingly, the patient was transferred to the delivery suite at 12 pm for further observation and management. The patient’s vital signs in both units upon arrival were stable, with blood pressures ranging from 97/70–106/75 mm Hg, and a stable pulse of around 98 bpm. At 12 pm, on cardiotocography, the patient had a reassuring tracing with irregular contractions. Twenty minutes after, the monitor displayed recurrent deep variable decelerations with a prolonged deceleration lasting around 5 minutes. The patient’s pelvic exam showed a closed, long and posterior cervix; however, blood pressure was on the low side (90/40 mm Hg). Resuscitative measures ensued that included 2 intravenous lines, hydration with crystalloids, and maternal oxygenation, as well as left lateral decubitus positioning. At 12:30 pm, despite the brief improvement in the patient’s tracing, a non-reassuring tracing predominated with fetal bradycardia. At this point, the maternal blood pressure was continuously dropping reaching 80/30 mm Hg, at which the patient reported sudden pleuritic chest pain, and the Rapid response team and Adult code team were called. At 12:45 pm, arterial blood gases were taken that demonstrated respiratory alkalosis; our differential at this point was pulmonary embolism versus pyelonephritis and rapidly progressing sepsis. At 12:50 pm, upon arrival of the rapid response team, the patient had a nadir blood pressure of 60/30 mm Hg that persisted despite vasopressors. Upon arrival, the code team immediately instituted resuscitative measures, initiating vasopressors. The patient was then intubated, despite stable oxygen saturation at 94%, due to worsening refractory hypotension and decreasing level of consciousness, and she was transferred to the intensive care unit, after placement of a central line.

In the intensive care unit, at 3:36 pm, the patient coded and CPR began. After 4 minutes of CPR, at 3:39 pm, a peri-mortem cesarean section was performed at bedside through a midline incision. At 3:40 pm, the baby was delivered, with Apgar’s 0 and 0 at 1 and 5 min, respectively. On abdominal entry, massive bleeding and clots were noted and the source was identified to be a ruptured splenic artery aneurysm. Massive transfusion protocol was activated; patient was packed and transferred to the operating room. In the operating room, the patient went on to have a splenectomy with temporary abdominal closure and correction of the coagulopathy. Intraoperatively, she received 14 units of packed red blood cells, 14 units of platelets and 3 units of fresh frozen plasma. The massive transfusion protocol was continued postoperatively. Around 24 hours postoperatively, the patient had an increased abdominal girth and pressure, with suspicion of abdominal compartment syndrome. In the operating room, she underwent an exploratory laparotomy, a left hemicolectomy for hematoma of the mesentery, and closure of pancreatic tail bleeding stump; she went on to have abdominal closure with a colostomy after 2 days. The patient’s postoperative course was complicated by multiorgan failure. Throughout her stay, she became anuric and underwent daily dialysis, a ventriculostomy was placed for Brain edema and hydrocephalus, and she progressed into liver failure with rising transaminases, and an incidental finding of hepatitis C infection. A tracheostomy was also placed around 2 weeks postoperatively that was complicated by recurrent bleeding, for which the patient went into cardiac arrest twice. During her ICU stay, she also contracted multidrug resistant Acinetobacter and fungal septicemia. In short, the patient received a total of 100 units of packed red blood cells in 3 days. She was discharged home after six months of hospital stay. Eventually, the tracheostomy was decannulated, the colostomy was reversed, and uterine dehiscence was repaired. After 1 year, the patient has slurred speech, hoarseness, and short term memory loss, and she is also undergoing physiotherapy for spasticity. Nonetheless, she is a survivor and a living example of what people can go through and survive.

## 3. Discussion

The first successful treatment of a ruptured splenic artery aneurysm was reported by McLeod in 1940 [27]. Due to the absence of controlled studies, management is based on the available observational studies, but there is no consensus guideline for management. However, taking into account elective operative mortality that ranges between 0.5 and 1.3% when compared to emergency repair, the general approach to splenic artery aneurysms is an early elective treatment when recognized, rather than watchful waiting, to minimize the risk of rupture [27]. The generally accepted guidelines include the elective repair of: symptomatic splenic artery aneurysm regardless of size, asymptomatic splenic artery aneurysm larger than 2 cm, and asymptomatic splenic artery aneurysm during pregnancy or in women of childbearing age [6]. The surgical options for the repair of splenic artery aneurysms vary from open to laparoscopic to embolization. In the pregnant patient with a symptomatic splenic artery aneurysm, the aim is immediate resuscitation via a cesarean laparotomy with splenectomy or splenopancreatectomy and ligation of the splenic artery [28]; one thing to keep in mind is that regardless of the approach, we should attempt to preserve the spleen; however, in some cases such as splenic devascularization, or aneurysmal adhesion to the pancreas, splenectomy, pancreatectomy or both may be necessary [28]. The surgical technique usually depends on the location of the SAA, with 80% located in the distal portion of the splenic artery [28].

The open surgical approach remains the gold standard approach for SAA repair, mainly when the aneurysm is located in the distal portion of the artery, or in the case of giant aneurysms where other approaches, such as endovascular treatment, would be suboptimal [29]. An alternative is the endovascular approach that has recently gained a lot of popularity with a variety of techniques and the advantage of a less invasive approach especially in patients that exhibit a high surgical risk; however, it is not without complications [30]. Also, there are no data on long term follow up of patients where the endovascular approach was used and there are no reports where these treatment modalities were used in ruptured SAA in pregnancy [30]. The laparoscopic approach is a safe therapeutic alternative for cases of elective splenic aneurysm repair; it carries the advantage of the rapid recovery, shorter hospital stay, and less postoperative pain compared with the open approach [31]. The laparoscopic approach is also suitable and safe in pregnant SAA patients. Compared with the open approach, laparoscopic splenic aneurysm repair has a lower risk of preterm labor in these patients due to minimal manipulation of intra-abdominal contents [31]. In fact, for stable pregnant women with an SAA ≥ 2 cm in diameter, the minimally invasive surgical intervention is the recommended method of treatment in the 1st or 2nd trimester [31].

‘An ounce of prevention is worth a pound of cure’ [Benjamin Franklin]; so, one can only question whether a means at preventing the high rates of morbidity and mortality associated with splenic artery aneurysm in pregnancy is feasible. According to a review that was conducted to ascertain a possible benefit of screening pregnant women for SAA, the authors concluded that “radiologic screening of all childbearing-aged females and routine screening during pregnancy are not warranted, but identification of those at greater risk of harboring an asymptomatic SAA, along with the early institution of treatment, according to current guidelines, may prevent maternal and fetal mortality in the rare event of SAA rupture during pregnancy [10].” The cost-benefit of screening all pregnant women for SAA is unfavorable due to the rarity of the disease [10]. Further studies are needed to determine which high risk population would benefit the most from screening, and to determine the most sensitive and cost-efficient radiologic method to detect asymptomatic SAA [10]. Routine screening of the splenic artery by ultrasound and doppler may be considered in selective pregnant patients with predisposing risk factors such as hypertension, multiparity, liver and pancreatic diseases, that predispose them to this morbid condition. To go back to our patient, relevant risk factors our patient had included a past obstetrical history significant for preeclampsia with severe features necessitating an indicated preterm delivery, and a past medical history significant for chronic hypertension, as well as hepatitis C that was discovered on incidental studies during her stay. In retrospect and keeping in mind all of the risk factors our patient had, one may have considered routine screening with an ultrasound, but then again ‘everything in retrospect is obvious [Michael M. Lewis].’ Another question at motive is when to deliver a deteriorating mother, and one can infer that, as in all cases, maternal stabilization should always take precedence prior to delivery. However, whenever the suspicion of intraabdominal bleeding is high, an emergency cesarean section should be performed. According to the American College of Obstetricians and Gynecologists (ACOG) and the American Academy of Pediatrics (AAP), an emergency cesarean section is that which is performed with a time frame of 30 minutes from decision for cesarean delivery to the start (incision) of the procedure. However, an emergency cesarean, with the largest category of indications being non-reassuring fetal heart rates, cannot easily be defined and it encompasses a broad range of acuity and severity [32]. Finally, a peri-mortem, or resuscitative hysterotomy, is defined as a cesarean section performed during active maternal cardiac arrest, with the primary goal aimed at maternal resuscitation, and a goal delivery time within the first 4 minutes of arrest [33].

To our best knowledge, this is the second case of splenic artery aneurysm rupture during pregnancy reported in Lebanon, with the previously reported case being encountered in a neighboring hospital within Beirut, less than a year ago [34]. However, in contrast to our case, Creidi et al. performed an aneusymectomy of her large SAA (15.5 cm vs. 3 cm), which was detected also at an earlier gestational interval than our patient’s (12+4 vs. 30+5 weeks of gestation) [34]. It is also well known that abdominal surgery is safer and less challenging when performed early in pregnancy as compared to a more advanced gestational age. Moreover, the larger nature of the their patient’s aneurysm (15.5 cm vs. 3 cm), allowed for prompt recognition with subsequent prophylactic and timely intervention.

## 4. Conclusion

In conclusion, SAA rupture in pregnancy is a rare but life-threatening obstetrical complication, associated with high rates of maternal and fetal morbidity and mortality. A high index of suspicion must be maintained while evaluating any pregnant woman with abdominal pain, especially in the setting of hemodynamic instability. While diagnostic modalities have proven worthy, with ultrasound being the preferred imaging method in pregnancy, the absence of controlled studies continues to surround management guidelines. Moreover, evidence substantiating the benefit of screening pregnant women for this high risk condition, as well as to determine the specific subgroup of childbearing-aged females that may benefit from the early institution of screening are still lacking and should be the focus of future research.
